# Loxl2 is a mediator of cardiac aging in *Drosophila melanogaster*, genetically examining the role of aging clock genes

**DOI:** 10.1093/g3journal/jkab381

**Published:** 2021-11-04

**Authors:** Mark J Bouska, Hua Bai

**Affiliations:** Department of Genetics, Development, & Cell Biology, Iowa State University, Ames, IA 50011, USA

**Keywords:** *Drosophila*, Loxl2, aging clocks, arrhythmia, lifespan, Cadherin, Pericardin

## Abstract

Transcriptomic, proteomic, and methylation aging clocks demonstrate that aging has a predictable preset program, while transcriptome trajectory turning points indicate that the 20–40 age range in humans is the likely stage at which the progressive loss of homeostatic control, and in turn aging, begins to have detrimental effects. Turning points in this age range overlapping with human aging clock genes revealed five candidates that we hypothesized could play a role in aging or age-related physiological decline. To examine these gene’s effects on lifespan and health-span, we utilized whole body and heart-specific gene knockdown of human orthologs in *Drosophila melanogaster*. Whole body lysyl oxidase like 2 (Loxl2), fz3, and Glo1 RNAi positively affected lifespan as did heart-specific Loxl2 knockdown. Loxl2 inhibition concurrently reduced age-related cardiac arrythmia and collagen (Pericardin) fiber width. Loxl2 binds several transcription factors in humans and RT-qPCR confirmed that a conserved transcriptional target *CDH1* (*Drosophila* CadN2) has expression levels which correlate with Loxl2 reduction in *Drosophila*. These results point to conserved pathways and multiple mechanisms by which inhibition of Loxl2 can be beneficial to heart health and organismal aging.

## Introduction

Molecular aging clocks use transcriptomics (Mamoshina *et al.* 2018; González-Velasco *et al.* 2020), proteomics (Lehallier *et al.* 2019; Johnson *et al.* 2020), and epigenetic markers (Horvath 2013; Bergsma and Rogaeva 2020; Galkin *et al.* 2021) to predict a subject’s age with high accuracy. In addition, studies examining the transcriptomes and proteomes during aging have shown that the majority of genes change their expression trends (transcriptome trajectory turning points) between ages 20 and 40 in multiple tissues (Somel *et al.* 2010; Skene *et al.* 2017; Lehallier *et al.* 2019). The age range of these expression turning points corresponds to the time at which most adults will begin to visibly detect aging phenotypes ranging from skin wrinkles to graying hair (Albert *et al.* 2007; Panhard *et al.* 2012). We, therefore, hypothesized that a number of the molecules having turning points in this age range should present in aging clock data, and play a role in aging or age-related phenotypes.

Through the examination of aging clocks (Horvath 2013; Lehallier *et al.* 2019) and transcriptome trajectory turning points (Skene *et al.* 2017), we identified Lysyl oxidase like 2 (*Loxl2*). Lysyl oxidases oxidatively deaminate lysine and hydroxylysine residues forming aldehydes on collagens which can then covalently bond, cross-linking the collagens and forming fibers (de Jong *et al.* 2011; Erasmus *et al.* 2020). Collagen is not only the most abundant protein in the extracellular matrix (Frantz *et al.* 2010), but when excessive fibrillar collagen accumulates in the heart it is recognized as cardiac fibrosis (Manabe *et al.* 2002) and the incidence of cardiac arrhythmia is frequently dependent on collagen texture (de Jong *et al.* 2011).

Loxl2 also binds transcription factors in the nucleus to inhibit and promote the transcription of genes that play a role in age-related diseases ranging from cancers to cardiovascular diseases (Wen *et al.* 2020). In human cells, Loxl2 binds the snail transcription factor to regulate E-Cadherin 1 (CDH1) expression (Peinado *et al.* 2005), and it has been demonstrated in flies that E-cadherin is dependent on the snail1 homolog escargot (esg; Tanaka-Matakatsu *et al.* 1996). Cadherins, Snail1, and LOXL2 are major components in the endothelial-to-mesenchymal transition (EMT; Cuevas *et al.* 2014) and EMT is a key modulator of age-related diseases including cancer and cardiovascular disease (Brabletz *et al.* 2018; Santos *et al.* 2019; Chen *et al.* 2020). Further linking Loxl2, cadherins, and collagen is the observation that the EMT program is highly correlated with epigenetic regulation that remodels the ECM. (Peixoto 2019). We therefore aimed to examine the multifaceted role of Loxl2 in aging and aging phenotypes using *Drosophila melanogaster*. 

## Materials and methods

### Lifespan assay

All assays were performed in females unless noted. Fly lines are listed in Supplementary Table S1. Flies were maintained at 25°, 60% relative humidity, and 12-h light/dark cycle in vials containing 25 adults. Larvae were reared on a standard cornmeal and yeast-based diet. The standard cornmeal diet consists of the following master mix of materials pumped into vials: water (1700 ml), agar (15.8 g), yeast (50 g), Cornmeal (104 g), sugar (220 g), TEGOSEPT (4.76 g), and 95% EtOH (18.4 ml). Five-day old adults received standard food with either RU486 mifepristone in 95% ethanol to induce RNAi (200 µM) or 95% ethanol in control food. Food vials were exchanged every 2–3 days. Calculations and chart were generated using Log Rank Mantel Cox test and JMP statistical discovery software. Graphs were generated using GraphPad Prism version 7.03.

### Heartbeat analysis

Semi-intact *Drosophila* adult fly hearts were dissected and imaged in oxygenated artificial hemolymph according to previously described protocols (Fink *et al.* 2009). Briefly, artificial hemolymph was prepared using NaCl (108 mM), KCl (5 mM), CaCl_2_·2H_2_O (2 mM), MgCl_2_·6H_2_O (8 mM), NaH_2_PO_4_ (1 mM), NaHCO_3_ (4 mM), Hepes pH 7.1 (15 mM), sucrose (10 mM), and Trehalose (5 mM). Heart recordings were performed at 100 frames per second for 30 s on each fly using HCImage software (Hamamatsu Corporation), Hamamatsu digital camera C11440, Olympus BX51WI microscope, at 10× magnification. Arrhythmia index was calculated using the standard deviation of heart periods (time between diastoles)/median period using Semi-automated Optical Heartbeat Analysis (SOHA) software (Fink *et al.* 2009).

### Fluorescent immunohistochemistry

Fly abdomens were dissected in 1× PBS, fixed in 4% paraformaldehyde diluted in 0.3% 1× Phosphate-buffered saline plus Triton X-100 (PBST) for 20–30 min. Samples were washed in PBST, blocked with PBST plus Donkey or Goat Serum Albumin for 1 h, and then washed in PBST three times for 10 min each. The samples were incubated with anti-Pericardin (Developmental Studies Hybridoma Bank # EC11) at 2 μg/ml overnight at 4°C, washed in PBST three times for 10 min each, then incubated with Alexa Fluor 594 anti-mouse IgG (Jackson ImmunoResearch # 115-585-166) for 2 h at room temperature. Samples were washed in PBST three times for 10 min each. Hoechst (Immunochemistry Technologies # 639) and Alexa Fluor 488 Phalloidin (Thermo Fisher scientific # 12379) were applied for 30 min followed by washing in 1× PBS and mounted in ProLong Diamond Antifade Mountant (Invitrogen # P36961) according to manufacturer’s protocols.

### Confocal microscopy and image analysis

Images were taken on an Olympus FV3000 confocal microscope. Filament widths were quantified with CellSens Software version 2.2. Images finalized using ImageJ Fiji v1.53c.

### Western blotting

Protein from 10 flies per replicate was extracted using TissueLyser II (Qiagen), Pierce IP lysis buffer, Protease Inhibitor Cocktail, and Phenylmethanesulfonyl Fluoride. Protein was quantified using Bicinchoninic acid assay (Thermo Fisher # 23225) on a Biotek Epoch 2 microplate spectrophotometer. Protein was denatured in 2-Mercaptoethanol and 2× Laemmli sample buffer at 95° for 5 min and loaded onto 4–20% Mini-PROTEAN TGX Precast Protein Gels (Bio-Rad Laboratories # 4561093) then rapid transfer to Polyvinylidene difluoride membranes (Bio Rad # 1620177). Membranes were blocked in 5% Bovine Serum Albumin (BSA) and 1× Tris-Buffered Saline plus Tween (TBST) for 1 h. Pericardin antibody at 0.2 μg/ml (Developmental Studies Hybridoma Bank# EC11) or B-tubulin at 0.2 μg/ml (DSBH # E7) were applied in 5% BSA at 4° overnight then washed in TBST four times for 5 min. Secondary anti-mouse horseradish peroxidase (HRP; Jackson ImmunoResearch # 115-035-174) or anti-rabbit HRP (Jackson ImmunoResearch # 711-035-152) were applied at 1:5000 in 1% BSA for 1 h at room temperature then washed in TBST four times for 5 min. Super Signal West Pico Plus Chemiluminescent Substrate (Thermo Scientific # 34577) was applied and then imaged with Bio-Rad Chemidoc. Stripping of the blot was performed using Restore Western Blot Stripping Buffer (Thermo Scientific # 21059). Band intensity ratios were calculated using BioRad Image Lab 6.1.

### RT-qPCR

RNA from 10 flies per replicate was extracted using TissueLyser II (Qiagen), Trizol, chloroform, and isopropanol, then washed in RNAse free ethanol. DNase treatment (Turbo DNA-free, Ambion # AM1907) was performed following manufacturer’s protocol. RNA was quantified using Thermo Scientific Nanodrop Lite Spectrophotometer. cDNA was synthesized using qScript cDNA Synthesis Kit (Quanta Bio # 95047-100) on the Applied Biosystems ProFlex PCR System. qPCR was performed using primers found in Supplementary Table S2 and PowerUp SYBR Green Master Mix (Thermo Fisher # A25742) on Applied Biosystems QuantStudio 3. Expression was calculated relative to Ribosomal protein L32 (RpL32).

## Results

### Overlapping genes in aging clocks

**Figure 1:**
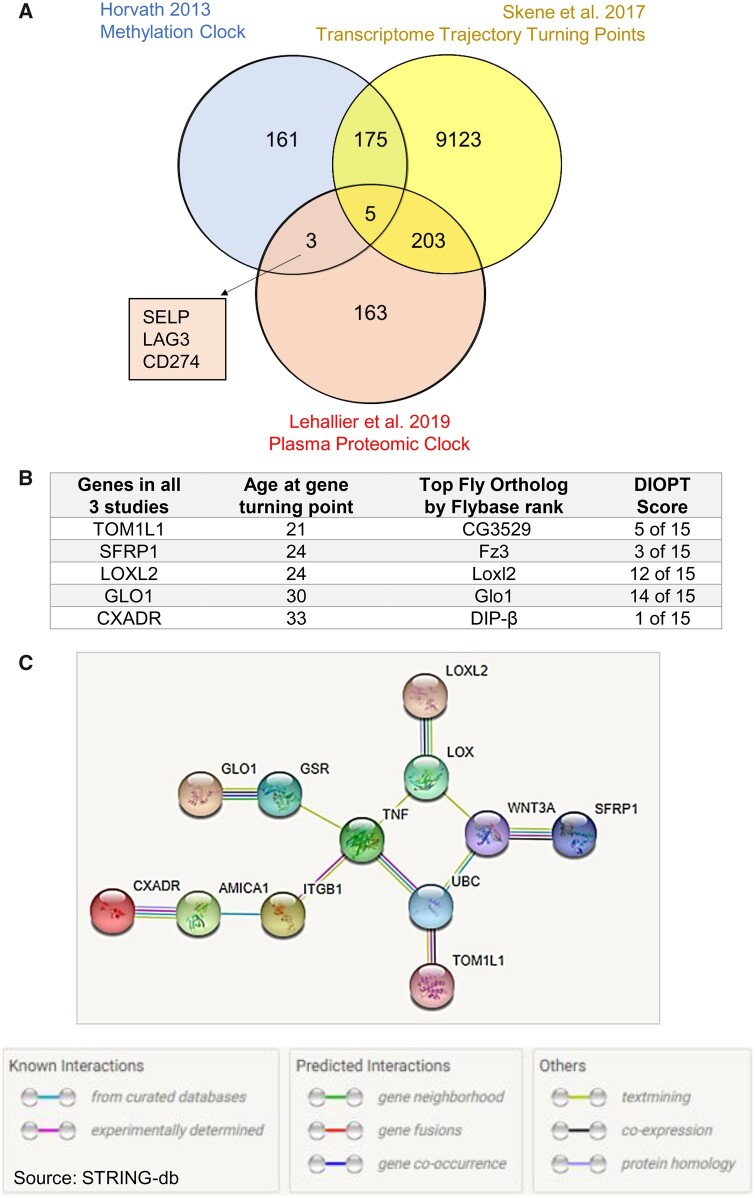
Overlapping genes in aging clocks. (A) Numbers of genes represented in the Horvath methylation epigenetic aging clock (Horvath 2013), Lehallier blood plasma proteomic clock (Lehallier *et al.* 2019), and genes that have transcriptome trajectory turning points occurring between ages 19 and 41 in the brain (Skene *et al.* 2017) including the amount of gene overlap between these studies. The identity of three genes not investigated in this study, but are found in the Horvath methylation epigenetic aging clock and Lehallier blood plasma proteomic clock, are found in the square inset. (B) The identity of the five human genes found in all three studies from 1A, the age at which they have human transcriptome trajectory turning points, their top ranking *Drosophila* ortholog, and their DRSC Integrative Ortholog Prediction Tool score (DIOPT version 9; Hu *et al.* 2011). (C) STRING-db analysis for how the five genes are connected in literature. The five genes are the outermost nodes.

We first compared the list of genes that make up the Horvath methylation epigenetic aging clock (Horvath 2013) with a blood plasma proteomic clock (Lehallier *et al.* 2019). We next selected genes that have transcriptome trajectory turning points occurring between ages 19 and 41 in the brain (Skene *et al.* 2017; Figure  1A). These ages were selected because the majority of transcriptome trajectory turning points, and an end of key developmental timepoints, occur within this window (Somel *et al.* 2010; Skene *et al.* 2017). Only five genes were found to overlap between the brain turning points, plasma proteomic clock, and the methylation clock; Target Of Myb1 Like 1 Membrane Trafficking Protein (*TOM1L1*), Secreted Frizzled Related Protein 1 (*SFRP1*), Loxl2, Glyoxalase I (*GLO1*), and CXADR Ig-Like Cell Adhesion Molecule (*CXADR*; Figure  1B).

Although at first glance these seem relatively unrelated, however, a gene network of the five genes in a STRING-db analysis suggests that the five genes may function around a TNF/WNT3A axis (Figure  1C). The genes are also target genes of the CAMP Responsive Element Binding Protein 1 transcription factor (Rouillard *et al.* 2016), possibly indicating a common regulatory axis.

We employed the model organism *D.* *melanogaster* due to its short lifespan and extensively available genetic toolkits to examine the age-related effects of these genes. We used Flybase (Larkin *et al.* 2021) to identify *Drosophila* genes with the highest ortholog scores for these five human genes (Figure  1B). Human CXADR had low homology with *Drosophila* proteins and was excluded from further study.

### Loxl2, fz3, and Glo1 reduction affects lifespan

**Figure 2:**
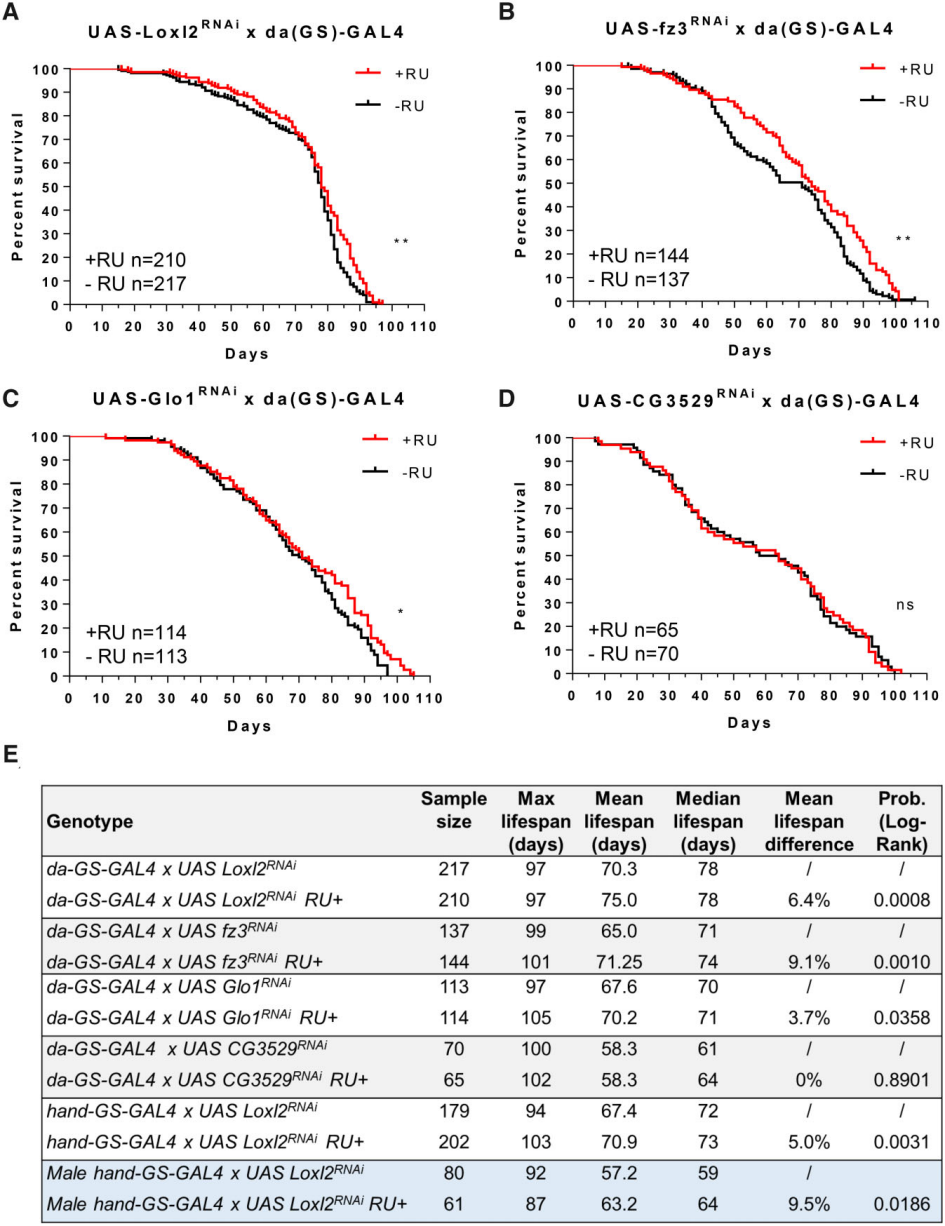
Loxl2, fz3, and Glo1 reduction affects lifespan. (A–D) Lifespan assays for UAS RNAi of Glo1, Loxl2, fz3, and CG3529 fly lines crossed with whole body *da*(GS)-GAL4 lines. RNAi was initiated (+RU *vs* control −RU) in day five adults and continued until death. (E) Summary of lifespan assays performed in this study using JMP statistical discovery software. Log Rank Mantel Cox test *P ≤ 0.05, **P ≤ 0.01, ***P ≤ 0.001, ****P ≤ 0.0001, ns, not significant.

To determine if the four genes may be relevant to *Drosophila* aging, UAS RNAi fly lines for the candidate genes were crossed with whole body daughterless-GeneSwitch GAL4 (*da*(GS)-GAL4) lines. RNAi was initiated by RU486 (mifepristone) chemical feeding in food starting on day five post-eclosion (to avoid impacting development) and continued until death. RNAi of Loxl2, fz3, and Glo1 significantly affected lifespan of female flies while CG3529 (TOM1L1 homolog) did not (Figure  2, A–D). Mean lifespan increased in the three fly lines by 3.7%, 6.4%, and 9.1%, respectively (Figure  2E).

### Loxl2 RNAi reduces Pericardin filament width with age

**Figure 3:**
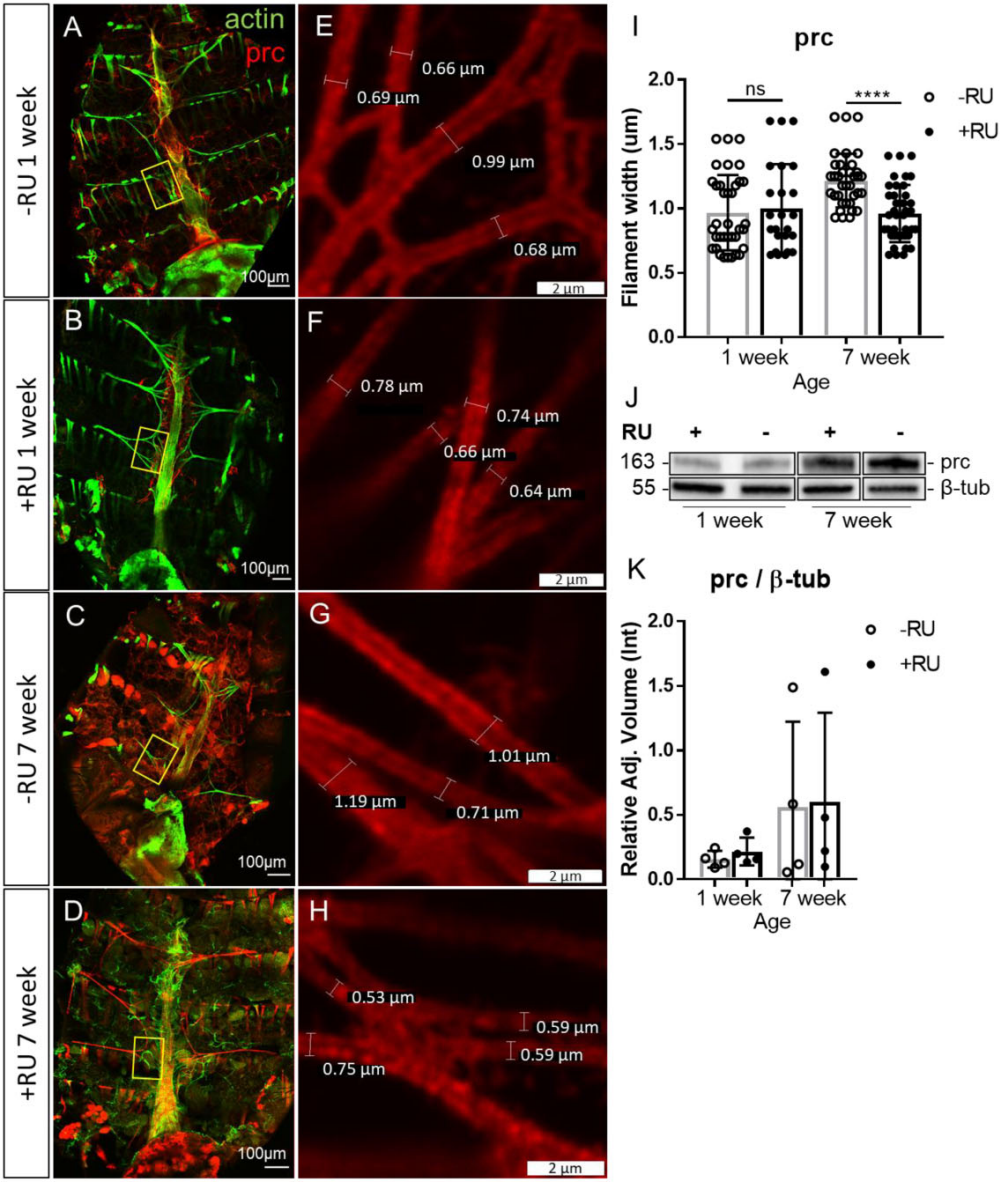
Loxl2 RNAi reduces Pericardin filament width with age. (A–D) Dissected *Drosophila* abdomen immuno-stained for Pericardin and stained with phalloidin to indicate actin filaments in alary and cardiac muscles of *da*(GS)-GAL4 × UAS-*Loxl2* RNAi flies +/−RU induction. Yellow boxes indicate the same region in which measurements were performed for all flies. (E–H) Magnification of representative Pericardin filament measurements. (I) Quantification of 4–5 filament widths for 5–7 flies from 1 to 7 weeks of age. Unpaired *t*-test *P ≤ 0.05, **P ≤ 0.01, ***P ≤ 0.001, ****P ≤ 0.0001, ns, not significant. (J) Representative Pericardin Western blots from 1 to 7 week flies. (K) Quantification of Pericardin Western blots from Figure  3J relative to β-tub; *n* = 4. Unpaired *t*-test *P ≤ 0.05, **P ≤ 0.01, ***P ≤ 0.001, ****P  ≤ 0.0001, ns, not significant.

Lysl oxidases function to deaminate collagen fibers, allowing their crosslinking (Erasmus *et al.* 2020). We, therefore, examined the effect of Loxl2 on *Drosophila* collagen IV; Pericardin. Pericardin (prc) levels and filament width have been shown to increase with age, and reducing collagen-interacting protein SPARC increases lifespan while its overexpression increases arrhythmicity (Vaughan *et al.* 2018). We immuno-stained for Pericardin (Figure  3, A–D) and measured Pericardin filament width in alary muscles near the heart tube (Figure  3, E–H). Quantification revealed that filament widths increased with age and RNAi for Loxl2 significantly reduced this effect (Figure  3I). Western blots against Pericardin were then performed to determine if the filament width may be a result of changing Pericardin levels (Figure  3J, Supplementary Figure S1), but no significant difference of Pericardin levels between age matched +/−RU fed lines was seen (Figure  3K).

### Expression of Loxl2 regulated genes from human age-related disorders

**Figure 4:**
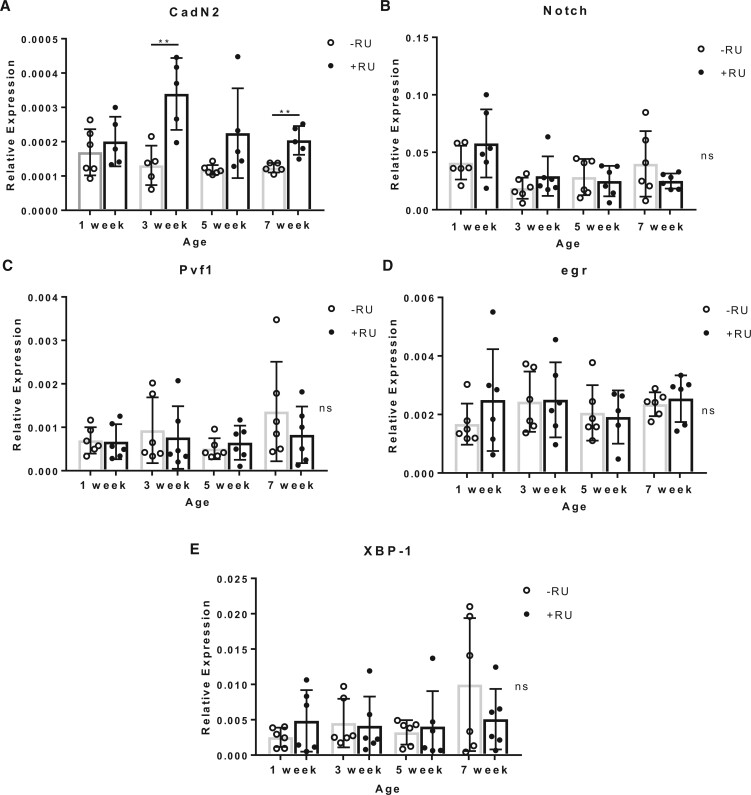
Expression of Loxl2 regulated genes from human age-related disorders. (A–E) RT-qPCR for 5–6 replicates of 10 whole *da*(GS)-GAL4 × UAS-*Loxl2* RNAi flies +/−RU induction (starting on day 5) from four time points in the *Drosophila* lifespan. CadN2 was significantly upregulated when Loxl2 is repressed at week three and seven; one outlier was removed from each sample before analysis. Unpaired *t*-test *P ≤ 0.05, **P ≤ 0.01, *** *P* ≤ 0.001, **** *P* ≤ 0.0001, ns, not significant.

Loxl2 has also been shown to have numerous noncollagen-related functions. Critically, Loxl2 maintains transcriptional regulation of genes that play a role in age-related diseases including cancers and cardiovascular disease genes (Wen *et al.* 2020). These genes include Tumor Necrosis Factor alpha (*TNFα*) [eiger; *egr*] (Schumacher and Prasad 2018; Rolski and Błyszczuk 2020), X-box binding protein 1 (*XBP-1*; Duan *et al.* 2015; Wang *et al.* 2019), Vascular endothelial growth factor A (*VEGF-A*) [PDGF- and VEGF-related factor 1; *Pvf-1*] (Taimeh *et al.* 2013; Braile *et al.* 2020), NOTCH1 (Garg *et al.* 2005), and Cadherin 1; *CDH1* [Cadherin-N2; *CadN2*] (Vite and Radice 2014; Mayosi *et al.* 2017; Turkowski *et al.* 2017); *Drosophila* homologs in square brackets (Larkin *et al.* 2021). We therefore asked if these genes have differential expression under Loxl2 RNAi with age (Figure  4, A–E). We found that CadN2 expression significantly increased at three and seven weeks under Loxl2 RNAi (Figure  4A).

### Loxl2 RNAi improves cardiac arrhythmia

Cadherins play a key role in cell matching during *Drosophila* heart development (Zhang *et al.* 2018). CadN2 is an underexplored protein in *Drosophila* and is the best matched ortholog to 17 Cadherins in humans (Larkin *et al.* 2021), of which three—*CDH2* (Vite and Radice 2014; Mayosi *et al.* 2017; Turkowski *et al.* 2017), *CDH5* (Bouwens *et al.* 2020), and *CDH13* (Teng *et al.* 2015; Verweij *et al.* 2017)—play a role in, or correlate with, cardiovascular disease. Furthermore, Loxl2 has been implicated in collagen-related cardiovascular fibrosis, in particular, its reduction can reduce not only cardiac fibrosis (Yang *et al.* 2016) but also vascular stiffening (Steppan *et al.* 2019) with age in mammals.

**Figure 5:**
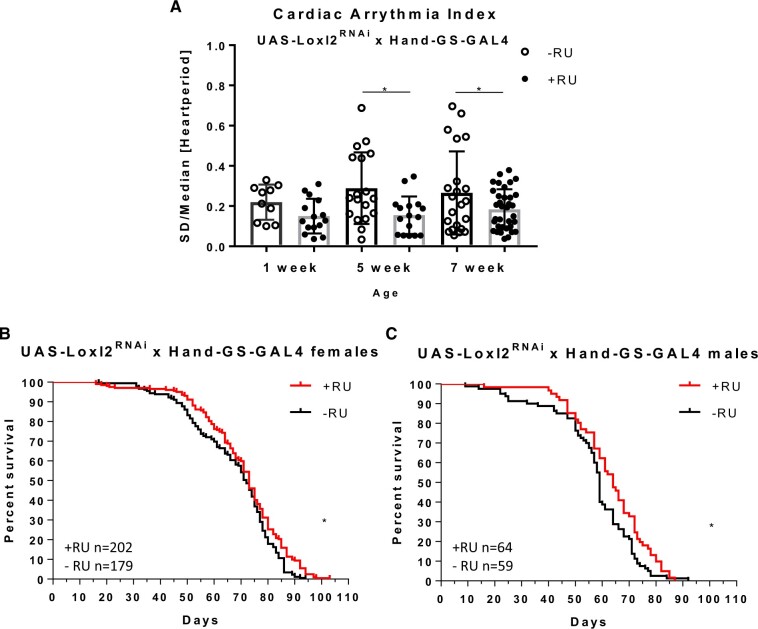
Loxl2 RNAi positively affects the heart with age. (A) Whole body knockdown using *da*(GS)-GAL4 crossed with UAS-*Loxl2* RNAi reduces cardiac arrythmia indices in *Drosophila*. Unpaired *t*-test *P ≤ 0.05, **P ≤ 0.01, ***P ≤ 0.001, ****P ≤ 0.0001. (B, C) Heart-specific knockdown using *Hand*(GS)-GAL4 crossed with UAS-Loxl2 RNAi positively affects lifespan. Log Rank Mantel Cox test *P ≤ 0.05, **P ≤ 0.01, ***P ≤ 0.001, ****P ≤ 0.0001, ns, not significant.

To test if Loxl2 reduction could affect the *Drosophila* heart we made use of the SOHA software (Ocorr *et al.* 2009), to measure cardiac arrhythmia indices; which can be used as measures of heart health and cardiac aging (Ocorr *et al.* 2007). SOHA on crosses of UAS-Loxl2 RNAi flies with whole body daughterless-GeneSwitch GAL4 (*da*(GS)-GAL4) and RU486 feeding starting on day five revealed that the arrythmia indices of knockdown flies significantly improved *vs.* controls at aged timepoints (Figure  5A).

Due of the benefit of Loxl2 RNAi on the heart, we employed a *Hand*-GeneSwitch GAL4 (*Hand*(GS)-GAL4) heart-specific driver cross with UAS-Loxl2 RNAi to test if the lifespan benefits may be partially derived from heart Loxl2. We saw a significant mean lifespan benefit in both male and female flies (Figure  5, B and C).

## Discussion

This work is the first to demonstrate that the reduction of Loxl2 has a protective mechanism in the *Drosophila* heart and extends mean lifespan, consistent with Loxl2’s role in mammalian cardiovascular disease (Yang *et al.* 2016; Steppan *et al.* 2019). We also show inhibiting Loxl2 can reduce Pericardin filament width and increase CadN2 expression. Our future work aims to decouple their respective contribution to the protective effects of Loxl2 reduction during aging.

Our data suggest that Loxl’s action as a transcriptional regulator may be conserved, which we can now explore in more detail utilizing the advantages of the *Drosophila* genetic system (McGuire *et al.* 2004; Allocca et al. 2018; Heigwer *et al.* 2018). Loxl2’s known interactions indicate that its role in aging could be through EMT which involves a transitional state that may be partially activated during aging (Brabletz *et al.* 2018) and interestingly EMT is even being pursued in age-related telomere shortening (Imran *et al.* 2021). Further investigation into the extent Loxl2 regulation in the ECM and EMT pathways in *Drosophila* plays with age, particularly the molecular partners and signaling of Loxl2 in the ECM and nucleus, can now be genetically explored in *Drosophila*. It is tempting to speculate that aging clocks and transcriptomic trajectory turning points may be picking up signals of the EMT process at a critical timepoint (20–40 years), the time at which aging phenotypes begin to be seen in humans.

The arrhythmia indices in aging Loxl2 RNAi flies without RU revealed roughly 1/3 of flies had increased arrythmia at aged timepoints revealing a biphasic distribution of arrhythmicity in the population. This is consistent with the variation that generally occurs with age, although it is not fully understood what causes this increase in variation between individuals with age (even when the population starts from the same or similar genotypes). It may be that the effects of epigenetic changes during development generate phenotypic and molecular variations that are exaggerated with age (Zhang *et al.* 2020). The reduction of Loxl2 may help delay certain transcriptional aspects of aging and in turn delay the effects of those variations.

Although we cannot totally rule out off target effects of the RNAi, according to the Harvard Transgenic RNAi Project led by Norbert Perrimon group, there were no off targets predicted for the RNAi line used (Perkins *et al.* 2015). We confirmed Loxl2 knockdown with RT-qPCR (Supplementary Figure S2), and the *Drosophila* RNAi phenotypes are consistent with the known crosslinking functions and cardiac effects in mouse Loxl2 knockdown models (Yang *et al.* 2016; Martínez Rodríguez and González 2019). It is also true that some drivers do have an effect on lifespan when combined with RU feeding in mated female flies (Landis *et al.* 2015), the GAL4 drivers utilized within this manuscript have been previously tested for cardiac arrythmia, lifespan, and stress impacts under RU feeding (Shaposhnikov *et al.* 2015; Cannon *et al.* 2017). Several lines of evidence including reduced lifespan or increased arrythmia under RU for several crosses from the aforementioned studies, as well as our male Loxl2 and female CG3529 lifespan data, provide support that these drivers are less likely to be affected by a positive sexually dimorphic effect of RU feeding on lifespan.

Because we saw cardiac aging related phenotypes in our Loxl2 data, but no turning point studies have been done on the heart, we wanted to see if Loxl2 had turning points in the human heart. We utilized the GTEx Portal [from The Genotype-Tissue Expression (GTEx) Project] to determine if Loxl2 had a transcriptome trajectory turning point in the human atria or ventricles within the timeframe that was seen in the brain or blood transcriptome trajectory turning point studies (Skene *et al.* 2017; Lehallier *et al.* 2019). Importantly, Loxl2 changes its average expression trajectory in the heart chambers in the 30–39 age decile consistent with the majority of transcriptome trajectory turning points seen in the blood study (Lehallier *et al.* 2019). Further turning point studies using human heart data would reveal other molecules important in cardiovascular aging.

## Data availability

Fly lines are available upon request. The authors affirm that all data necessary for confirming the conclusions of this article are represented fully within the article, its tables, and figures.

Supplementary material is available at *G3* online.

## Supplementary Material

jkab381_Supplementary_Figures

jkab381_Supplementary_Tables
