# Supplementary material for: Loxl2 is a mediator of cardiac aging in Drosophila melanogaster, genetically examining the role of aging clock genes
Source: G3 (Bethesda). 2021 Nov 4;12(1):jkab381. doi: 10.1093/g3journal/jkab381 (PMC8727986; doi:10.1093/g3journal/jkab381)
Supplement: jkab381_Supplementary_Figures [file jkab381_supplementary_figures.zip › GENETICS-G3-2021-402989-s04.docx]

**Figure S1. Full Immunoblots from Figure 3J**. (A-D) Each prc western blot is in a box with its corresponding loading control (β-tub) image.

**Figure S2. Loxl2 RT-qPCR** (A) RT-qPCR of whole body knockdown using da(GS)-GAL4 crossed with UAS-Loxl2 RNAi comparing age matched RU feeding vs no RU control confirmed relative Loxl2 expression decreased under RU feeding. Unpaired t-test * p ≤ 0.05, ** p ≤ 0.01, *** p ≤ 0.001,**** p ≤ 0.0001, ns: not significant.
