# Supplementary figures and images for: Loxl2 is a mediator of cardiac aging in Drosophila melanogaster, genetically examining the role of aging clock genes
Source: G3 (Bethesda). 2021 Nov 4;12(1):jkab381. doi: 10.1093/g3journal/jkab381 (PMC8727986; doi:10.1093/g3journal/jkab381)

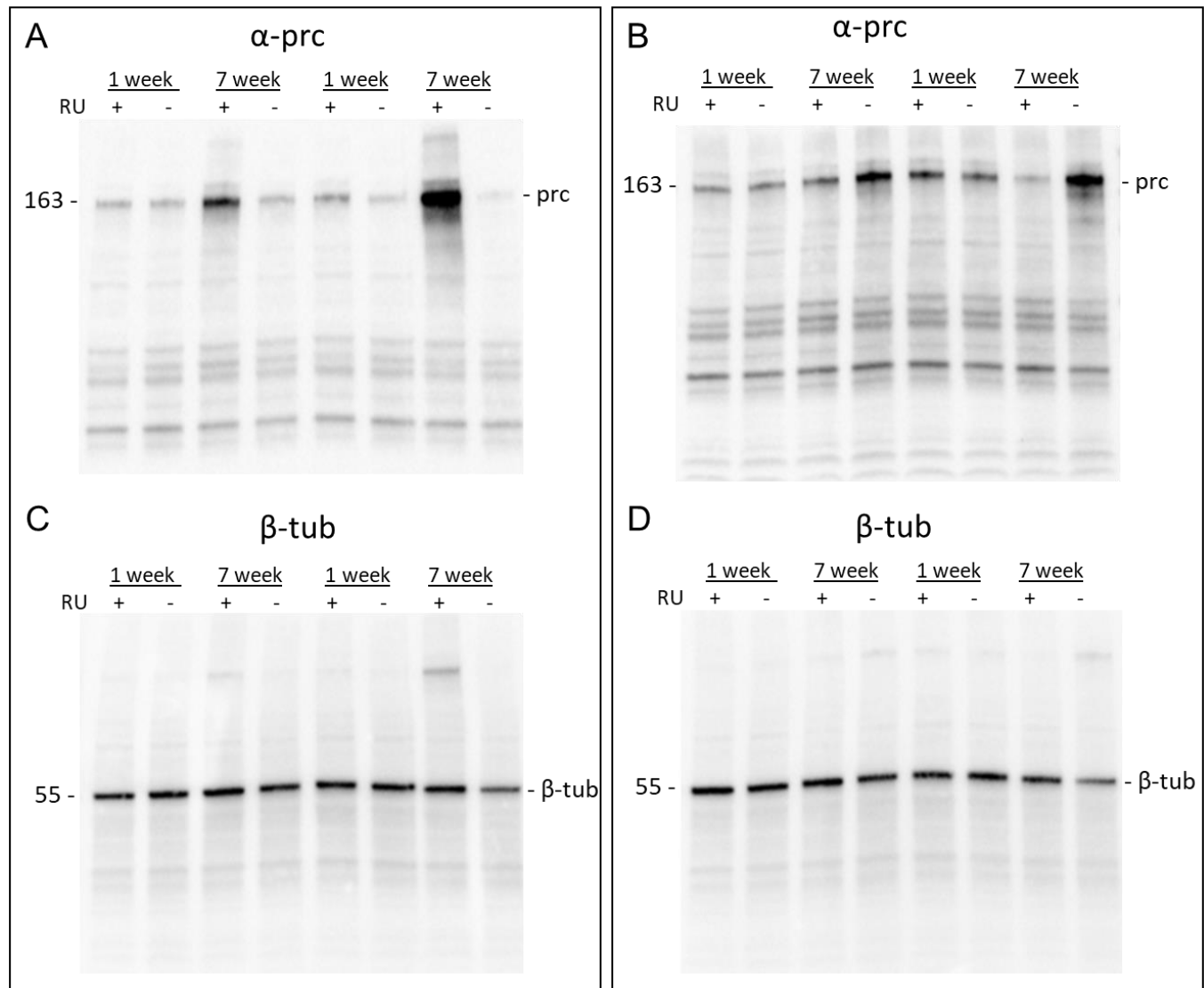

Supplement: jkab381_Supplementary_Figures [file jkab381_supplementary_figures.zip › GENETICS-G3-2021-402989-s01.pdf]

A

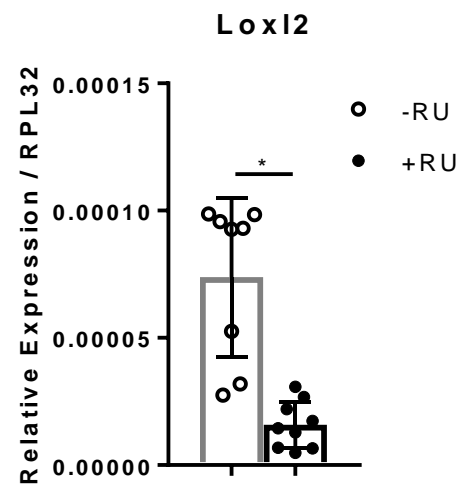

Supplement: jkab381_Supplementary_Figures [file jkab381_supplementary_figures.zip › GENETICS-G3-2021-402989-s02.pdf]
