# Supplementary material for: Loxl2 is a mediator of cardiac aging in Drosophila melanogaster, genetically examining the role of aging clock genes
Source: G3 (Bethesda). 2021 Nov 4;12(1):jkab381. doi: 10.1093/g3journal/jkab381 (PMC8727986; doi:10.1093/g3journal/jkab381)
Supplement: jkab381_Supplementary_Tables [file jkab381_supplementary_tables.docx]

**Table S1 Fly lines used in this study**

| **Bloomington *Drosophila* Stock Center#** | **Gene**  **Annotation symbol** | **Flybase**  **gene #** | **Gene** | **Genotype** | **Description** |
| --- | --- | --- | --- | --- | --- |
| 66951 | CG16785 | FBgn  0027343 | fz3 | y[1] sc[*] v[1] sev[21]; P{y[+t7.7] v[+t1.8]=TRiP.HMS05417}attP40 | Expresses dsRNA for RNAi of fz3 (FBgn0027343) under UAS control in the VALIUM20 vector. |
| 64867 | CG4402 | FBgn  0034660 | loxl2 | y[1] sc[*] v[1] sev[21]; P{y[+t7.7] v[+t1.8]=TRiP.HMC05740}attP40 | Expresses dsRNA for RNAi of Loxl2 (FBgn0034660) under UAS control in the VALIUM20 vector. |
| 53008 | CG3529 | FBgn  0035995 | CG3529 | y[1] v[1]; P{y[+t7.7] v[+t1.8]=TRiP.HMJ21726}attP40 | Expresses dsRNA for RNAi of CG3529 (FBgn0035995) under UAS control in the VALIUM20 vector. |
| 67297 | CG1707 | FBgn  0283450 | Glo1 | y[1] sc[*] v[1] sev[21]; P{y[+t7.7] v[+t1.8]=TRiP.HMC06400}attP40 | Expresses dsRNA for RNAi of Glo1 (FBgn0283450) under UAS control in the VALIUM20 vector. |
| **Gal4 line source reference** | | | | **Genotype** | **Description** |
| Tricoire et al. 2009 | | | | da(GS)-GAL4 | daughterless gene switch GAL4; Insertion is located on 2nd Chromosome |
| Monnier et al. 2012 | | | | Hand(GS)-GAL4 |  |

**Table S2 Primers used in this study**

| **Forward or Reverse 5'-3' sequence** | **Primer Name** | **Gene** | **Flybase ID #** | **Product size(bp)** |
| --- | --- | --- | --- | --- |
| CGCTTCTGGATGGACAACCT | Loxl2-f | Loxl2 | FBgn0034660 | 102 |
| CGCCTCTCCTGGTTCACAAT | Loxl2-r | Loxl2 | FBgn0034660 | 102 |
| AAAAGCAGCGTAGAACGCAC | SoxN-f | SoxN | FBgn0029123 | 108 |
| AGAAGCGGGAACACCGAAAA | SoxN-r | SoxN | FBgn0029123 | 108 |
| TTAATTATGGCCCCACATGCT | N-f | N (Notch) | FBgn0004647 | 109 |
| CGATGCGGTGTACGGTCTAA | N-r | N (Notch) | FBgn0004647 | 109 |
| GAGCAGGCCGAGAACAAGTAT | Pvf1-f | Pvf1 | FBgn0030964 | 135 |
| GTCCTATCGGGAGCCACTCA | Pvf1-r | Pvf1 | FBgn0030964 | 135 |
| GCAACTTCCAAAGAGAGCCC | egr-f | egr (TNFA) | FBgn0033483 | 114 |
| GTCCTCGGATCTGGCTGAAA | egr-r | egr (TNFA) | FBgn0033483 | 114 |
| CCAAGAACTCGCCGAACTGA | Xbp1-f | Xbp1 | FBgn0021872 | 121 |
| ATACCCTGCGGCAGATCCAA | Xbp1-r | Xbp1 | FBgn0021872 | 121 |
| ATCAAAATGGGCTGGCAACA | CadN2 | CadN2 | FBgn0262018 | 100 |
| GTGTTTGGGGAATCGGACAG | CadN2 | CadN2 | FBgn0262018 | 100 |
| AAGAAGCGCACCAAGCACTTCATC | RpL32-f | RpL32 | FBgn0002626 | 107 |
| TCTGTTGTCGATACCCTTGGGCTT | RpL32-r | RpL32 | FBgn0002626 | 107 |
